# Hysterical Paroxysmal Œdema
1Read at a Meeting of the Bristol Medico-Chirurgical Society on February 9th, 1898.


**Published:** 1898-09

**Authors:** F. H. Edgeworth

**Affiliations:** Assistant-Physician to the Bristol Royal Infirmary


### HYSTERICAL PAROXYSMAL ŒDEMA.[1]

BY

F. H. Edgeworth, M.B., B.C. Cantab., B.Sc. Lond.,

*Assistant-Physician to the Bristol Royal Infirmary.*

---

So little is known about the somewhat rare disease paroxysmal œdema, of either hysterical or other origin, that individual cases are worthy of record, insomuch as discussion may tend to elucidate the clinical symptoms and nature of the affection.

T. V. R., aged 24, by trade a plumber, came to the Bristol Royal Infirmary on June 9th, 1896, complaining of swelling of the left arm and left foot. On enquiry the following was obtained. There was no family history of any similar affection, to which the patient had become liable eleven years before. What would happen was this: he might go to bed perfectly well, to wake in the morning with a swollen foot, the swelling being so great that he could not put on his boot. This would last all day, and gradually subside. There was a burning, itching pain in the slightly red, swollen part, which could not be indented by a finger, though this was possible during its subsidence. These attacks were of fairly frequent occurrence; rarely did a month pass without one, and of late the interval between successive attacks had gradually lessened until, as a rule, it was not greater than a week. Any part of the body might be affected, sometimes the foot or leg, sometimes the arm or hand, often the face, rarely the trunk. If the upper part of the face were affected, he could not see out of the eye for several hours because of the swelling. Generally not more than one part of the body was affected at one time; if two at once, they were, as a rule, on the same side. But for the attacks he was in the best of health, and could not remember ever having had any other illness.

On examination, the patient looked a most healthy young man. The thoracic and abdominal organs were sound, and the urine was normal. The parts affected by the attack of œdema for which he came were the left forearm and the left foot. The left foot was uniformly swollen up to the level of the ankle, the swelling having precisely the same limits as a boot; it was slightly red, felt a little hot, and could be indented by the pressure of a finger. (The patient said that this attack was subsiding.) The left forearm was similarly affected

[1] Read at a Meeting of the Bristol Medico-Chirurgical Society on February 9th, 1898.

from two inches above the wrist to the level of the elbow; it extended right round the limb and had a circular margin above and below. In both the foot and forearm the skin seemed to be raised about a quarter of an inch above its normal level, and the margin of the œdematous area subsided to the level of the healthy skin in the space of about half an inch. There was no anæsthesia, analgesia, or thermo-anæsthesia of the skin in either the affected or normal parts. No hysterogenic zones were present. The muscular system was normal, as were also the skin-reflexes and tendon-jerks.

Several subsequent attacks were seen. Thus on June 21st the patient came with a swelling of the right hand, which had been present for nineteen hours. There was œdema of the hand and front and back of the wrist to two inches above the level of the joint. It pitted on pressure, and felt hot to the touch.

The most interesting attack, however, was witnessed on June 26th; it began with swelling of the left side of the upper lip, and gradually spread to the lower lip and left side of the face. On examination, there was seen swelling of the left side of the upper lip, the whole of the lower lip, and the left side of the face, gradually lessening upwards so that the circumorbital tissues were not affected. There was no hyperæmia of the skin, which nevertheless felt a little hotter than on the opposite side. The whole cheek, not merely the subcutaneous tissues, seemed swollen. The mucous membrane of the left cheek within the mouth presented numerous blebs, varying in size from a pea to a sixpenny-piece, filled with clear yellow serum. (On enquiry, the patient said that whenever the cheek was affected he always had blebs inside the mouth.) Other attacks were also seen—in the upper part of the face on the right side, almost closing the eye; in the right foot; in the right hand; and in the right breast: this last had a more or less circular form, and measured five inches across. No disturbances of sensation were found in any of the attacks. After a few weeks the patient was put on arsenic in increasing doses. Whilst under the influence of this drug the attacks became less acute and at greater intervals, and on August 21st the patient had not had any attack for four weeks and ceased attending.

The case was somewhat of a problem in diagnosis,—the long duration of the malady, its paroxysmal character, the absence of any obvious exciting cause, and the nature of the lesion led one to think that it must be of nervous origin. This opinion had its foundation in the well-known fact that it can be shown experimentally that the nervous system has a controlling influence over the permeability of the walls of the blood-vessels. The seat of origin of the discharge could hardly be the grey matter of the spinal cord, as the distribution of the lesions did not harmonise with either the peripheral nerve distribution, or the segmental skin-supply as determined by Thorburn, Head, and others.

But on reviewing the distribution of the areas of œdema, it was obvious that they were very similar to those affected by alterations in sensibility in some cases of hysteria. It was concluded that this case of paroxysmal œdema was a vaso-motor neurosis, of cerebral and probably cortical origin. Further than this it was scarcely possible to go.

Five months later, a second case appeared which carried these conclusions a step farther.

E. L., aged 22, a tailor's presser, came to the Bristol Royal Infirmary on November 6th, 1896, complaining of swelling of the right hand. He said that his illness began twelve months before, with a swelling in the sole of the right foot, which lasted a few hours only. Since then scarcely a week had gone by without one of the hands, or hand and forearm, or feet, becoming affected. As a rule, there was a little itching of the skin before the occurrence of the swelling, which latter persisted for about twenty-four hours and then gradually subsided. He otherwise enjoyed good health.

The patient was a flaxen-haired, healthy-looking young man ; the chest, abdomen, and urine were normal. The right hand was œdematous up to the level of the wrist, with a circular upper margin ; the affected area could scarcely be indented by firm pressure with a finger. The skin was not hyperæmic, nor was any increased heat appreciable. The important point about the case was that the skin affected by the œdema and for two inches above (*i.e.*, the area which would be covered by a high-buttoned glove) was partially anæsthetic to touch, partially anæsthetic to heat and cold, and totally analgesic to the prick of a pin. The nervous system was otherwise normal. Sensation was elsewhere perfect, movement was unimpaired, and the skin-reflexes and tendon-jerks normal. No hysterogenic zones were present.

On comparing these two cases, it was obvious that they were alike in some, dissimilar in other, features. The resemblances were in the nature of the lesion and the distribution and shape of the areas affected. The difference was that in the latter case there was an accompanying disturbance of sensation, which could not be accounted for by the œdematous condition of the skin (as was shown especially by its extension above the œdematous area), and which was of definite hysterical type.

A third case has recently come under observation.

Mrs. A. B., aged 37, came to the Royal Infirmary on February 1st, 1898, complaining that during the previous fortnight she had been troubled by the left arm swelling at night ; she would go to bed quite well, and be awakened between 2 and 3 a.m. by a numb pain in the left arm, and find that the arm was swollen from the shoulder downwards, slightly hot, not red, and feeling heavy. After a time she would fall asleep again, and wake in the morning with but little trace of the swelling left. This occurred two or three times a week. On examination, the only abnormality found was that in the left arm, from two inches above the elbow to two inches below the shoulder-joint, with a sharply defined circular upper and lower limit, there was an almost complete analgesia and thermo-anæsthesia, whilst tactile sensibility was normal.

These three cases show that paroxysmal œdema may be of hysterical origin.

Hysteria has long been recognised as an occasional cause of œdema; Sydenham first described it, and numerous instances have been recorded in more recent years. It is rarely an isolated phenomenon, and generally coexists with an arthralgia, paralysis, or contracture. The œdema is a hard one, *i.e.* can only be indented with difficulty. The colour of the outlying skin is variable, generally white, though occasionally bluish, as in the famous cases described by Charcot. The surface may be hyperæsthetic or anæsthetic. While its duration is variable, it is generally a most persistent phenomenon and may last for years.

The cases described above differ from these, more especially in their paroxysmal type, though otherwise they are similar. But the nature of the affection is, on further consideration, nct quite certain. Within the last few years a disease has been differentiated, to which various names have been given, the chief of which are paroxysmal œdema, vaso-motor œdema, angio-neurotic œdema, and sometimes Quincke's œdema after its first observer. Osler[1] has described some cases, and given a good description of the affection. It is a disease characterised by the occurrence of local œdematous swellings, more or less limited in extent and of transient duration. Severe colic is frequently associated with each attack. There is often a marked hereditary disposition; for instance, the disease was traced through five generations of one family. Its pathology is quite unknown, though Quincke and the majority of later observers consider it to be a vaso-motor neurosis.

The relation of the cases described above to Quincke's œdema is doubtful. They differ in the absence of hereditary history, and of associated colic, and in the presence of associated disturbances in sensation (cases 2 and 3), though the local phenomena

[1] *Am. J. M. Sc.*, 1888, xcv. 362.

are identical. It is noteworthy that in the first case the face was occasionally the seat of the affection, and if the cheek were attacked blebs appeared on the mucous membrane within the mouth. Now, in the recorded cases of hysterical œdema neither the face nor mouth has been affected, whilst it is well known to occur in Quincke's œdema. From this point of view, the first case would appear to be one of Quincke's œdema. On the other hand, the shape and distribution of the areas were exactly similar to those met with in hysterical disturbances of sensation and to those of the second case, and it can hardly be doubted that the latter one was of hysterical origin.

These considerations suggest that there exists a disease, paroxysmal œdema—a vaso-motor neurosis. In some cases (*e.g.* case 1) the shape and distribution of the œdematous areas suggest a cortical origin. Such cases may present disturbances in sensation of type similar to that met with in hysteria (cases 2 and 3). But it is doubtful whether all cases of paroxysmal œdema will fall under this head, for though the distribution of the areas affected is not sufficiently detailed in the recorded cases to answer the question with a decided negative, yet it is to be remarked that Osler does not mention any hysterical disturbances in the many cases of the family mentioned above. Apparently the two series of cases—those of hysterical type and those in which there is no such suggestive evidence—overlap, as is well shown by the first case recorded above. An important practical conclusion is that arsenic in large doses is worthy of a trial—in the first case it appeared to cure an affection which had lasted eleven years. This is the more noteworthy as no successful treatment has hitherto been proposed.

---

After the reading of Dr. Edgeworth's paper, Dr. MICHELL CLARKE said that these cases of paroxysmal œdema were very interesting, and at the same time difficult to explain. There appeared to be a series with at one end instances of localised œdema of limited extent occurring paroxysmally in one part of the body, with an intermediate group in which the œdema had a wider distribution and was attended with more or less marked sensory disturbances over the same area to those cases in which œdema accompanied well-marked hysterical affections, either sensory or motor. Probably cases of giant urticaria belonged to the same group of œdemas. The œdema occurring in well-developed hysterical affections had its best analogue in that experimentally produced during hypnosis, and might also be compared with the persistent slight œdema occasionally seen in cases of hemiplegia due to organic brain disease and then generally accompanied by much pain and tenderness in the paralysed limbs.

### FIFTY CONSECUTIVE INTRA-ABDOMINAL OPERATIONS.

